# Gastric adenocarcinoma of the fundic gland type: clinicopathological features of eight patients treated with endoscopic submucosal dissection

**DOI:** 10.1186/s13000-020-01047-2

**Published:** 2020-10-23

**Authors:** Chengfang Li, Xinglong Wu, Shuang Yang, Xiaorong Yang, Jin Yao, Hong Zheng

**Affiliations:** Department of Pathology, Affiliated Hospital of Zunyi Medical University, Guizhou, 563003 China

**Keywords:** Gastric adenocarcinoma of the fundic gland type, Chief cells, Pepsinogen-I, Endoscopic submucosal dissection, Pathological diagnosis, Endoscopic diagnosis

## Abstract

**Background:**

Gastric adenocarcinoma of the fundic gland type (GA-FG) has been added to the 2019 edition of the World Health Organization’s list of digestive system-associated cancers. This lesion differentiates toward the fundic gland and mostly involves chief cell-predominant differentiation with low-grade cytology. Clinicians and pathologists are still unaware of this rare disease; consequently, some cases are incorrectly diagnosed. This study aimed to investigate the clinicopathological features of GA-FG using retrospective analyses of endoscopic and pathological findings.

**Materials and methods:**

Samples were collected from patients diagnosed with GA-FG. The clinical courses of all patients were monitored prospectively and reviewed retrospectively. Available clinical information, endoscopic features, pathological appearance, and follow-up data were assessed. Immunohistochemistry [mucin (MUC) 2, MUC5, MUC6, P53, CDX2, Ki67, SYN, CD56, CGA, β-catenin, and pepsinogen-I] was examined using Envision two-step method.

**Results:**

Eight cases of endoscopic submucosal dissection (ESD) were obtained from our institution. Patient age ranged from 48 to 80 years (mean, 65 years). Some patients were on acid-suppressing medication. Most lesions were located in the upper third (*n* = 7) and one was in the middle third of the stomach. Six lesions were of the superficial flat type, whereas two were of the superficial elevated type. Narrow-band imaging using magnifying endoscopy showed irregular microvascular patterns (MVPs) in four cases and regular MVPs in the remaining cases. All lesions were primarily solitary and ~ 6 mm in diameter (largest, 12 mm). The main body of the tumors were localized in the mucosal layer, of which six cases invade into the submucosal layer. Well-formed glands of chief cells were predominant. Tumor cells were positive for pepsinogen-I, MUC6, SYN, and CD56. Lymphatic and vascular infiltration and metastatic and recurrent disease were not observed in any case.

**Conclusion:**

GA-FG, a well-differentiated adenocarcinoma with mild atypia, can be completely removed using ESD, with a favorable prognosis in patients.

## Introduction

Gastric adenocarcinoma of the fundic gland type (GA-FG), a new rare variant of gastric adenocarcinoma, presents with atypical cells with differentiation toward the fundic gland. According to the 5th edition of the World Health Organization’s (WHO 2019) classification of digestive system tumors, the characteristic oxyntic gland differentiation in GA-FG can be divided into three subcategories on the basis of the tumor composition, namely, chief cell predominant (~ 99% of reported cases), parietal cell predominant, and mixed phenotype [1]. Tsukamoto was the first to report a case of adenocarcinoma with chief-cell differentiation, which was named “gastric adenocarcinoma of fundic gland mucosa type” in 2007 [2]. Subsequently, Ueyama proposed a new histological type of gastric cancer with differentiation toward the fundic gland, named “gastric adenocarcinoma of the fundic gland type” in 2010 [3]. In total, 112 cases had already been reported in English literature when the concept of GA-FG was first proposed [4, 5]. The majority of GA-FG cases were observed in Asia (South Korea and Japan); however, cases were rare in other regions [6]. This difference may be owing to geographical reasons or lack of awareness. GA-FG accounts for 1% of patients with early gastric carcinoma who underwent esophagogastroduodenoscopy [7].

Although awareness regarding GA-FG is gradually increasing, few cases remain undiagnosed owing to the difficulty in arriving at a correct diagnosis. Endoscopically, GA-FG is classified into two categories, namely, submucosal tumor shape (superficial elevated type) (60%) and flat or depressed type (40%). The most common features of submucosal tumors are their whitish appearance, dilated vessels with branching architecture, and background mucosa without atrophic changes [7]. The histological appearance of GA-FG is often that of a well-differentiated neoplasm, with a tumor-bearing resemblance to that of the fundic glands. Furthermore, at low magnification, GA-FG, especially of the mixed cell type, can mimic a fundic gland polyp or a pyloric gland neoplasm [6]. It is necessary to differentiate GA-FG from fundic gland adenoma and other well-differentiated GA [4]. Histopathological examination is necessary for accurate diagnosis as it is difficult to distinguish lesions from GA-FG using endoscopy.

The molecular characteristics of most GA-FG samples involve nuclear β-catenin positivity in immunohistochemistry (IHC). Activation of the WNT-β-catenin signaling pathway is believed to play a role in tumorigenesis [8], although further studies are required to confirm this. Most GA-FGs were free from *Helicobacter pylori* infection, which was different from that observed in conventional gastric adenocarcinoma. This may be related to the use of acid-secretion inhibitors [9]; however, patients in most studies were not receiving medication. Here, we highlight our current understanding of GA-FG diagnosis, which involves a combination of endoscopic features, histological features, and the results of immunohistochemical staining, along with the medical history of patients.

## Materials and methods

In this study, samples were collected from eight Chinese patients diagnosed with GA-FG who visited the Affiliated Hospital of Zunyi Medical University during a 3-year-period from 2017 to 2019. The clinical courses of all patients were monitored prospectively and reviewed retrospectively. Available clinical information (including gender, age, medication history, and site and size of the lesion), endoscopic features [including shape and microvascular pattern (MVP)], pathological appearance (including histological and immunohistochemical data), and follow-up data were assessed.

Prior to endoscopic submucosal dissection (ESD), upper gastrointestinal endoscopy was performed via narrow-band imaging using magnifying endoscopy (NBI-ME) in addition to the conventional white-light endoscopy. All eight biopsy specimens were used for histological diagnosis.

Histopathological reviews were conducted by three pathologists based on the following: histological subcategories (chief cell predominant, parietal cell predominant, and mixed phenotype), architectural patterns, presence of cytonuclear atypia, depth involvement, and mucosal atrophy or intestinal metaplasia of adjacent mucosa. Architectural patterns noted included the presence of clustered/solid glands with or without well-formed glands, anastomosing cords, dilated glands, complex glands with multiple layers of cells, and cribriform glands [10]. Mucin (MUC) 2, MUC5, MUC6, P53, CDX2, Ki67, SYN, CD56, CGA, β-catenin, and pepsinogen-I were used as immunohistochemical markers and immunohistochemical staining was performed as per the manufacturer’s instructions.

Based on the above parameters, we summarize the cardinal histopathologic features to diagnose GA-FG as follows: (1) GA-FG arise most commonly from the normal gastric mucosa of the fundic gland region without intestinal metaplasia; (2) the lesion is almost invariably lined on the surface with normal-appearing foveolar-type epithelium; (3) the lesion differentiates toward the fundic gland and mostly involves chief cell-predominant differentiation; (4) the lesion demonstrates a complex architectural pattern of glands: anastomosing cords, dilated glands, cribriform glands; the atypia of the tumor cell is usually mild; (5) the lesion often invade the submucosal layer.

## Results

Clinicopathological details of all patients are summarized in Tables 1 and 2. Patient age ranged from 48 to 80 years, with an average age of 65 years. There were five female and three male patients. The lesions were located in the upper (*n* = 7) and middle third (*n* = 1) of the stomach.

**Table 1:** Clinical features in eight patients with GA-FG by ESD

Patient	Sex/age(y)	Clinical presentation	Medication	Location in stomach	HP infection	Macroscopic features	ME-NBI (MVP)	Endoscopic diagnosis	Follow up (months)
1	F/70	Reflux	PPI	fundus	–	Type0-IIa	irregular	NEN	NED (29)
2	F/74	Reflux	PPI	fundus	–	Type0-IIa	regular	Adenoma	NED (18)
3	M/80	Reflux	No	fundus	–	Type0-IIa	regular	GA-FG	NED (17)
4	F/51	Reflux	No	fundus	–	Type0-IIa	regular	NEN	NED (15)
5	F/59	Bloating	H2	Gastric body	–	Type0-IIc	irregular	Adenocarcinoma	NED (8)
6	M/72	Abdominal pain	PPI	fundus	–	Type0-IIa	irregular	GA-FG	NED (7)
7	F/48	Abdominal pain	No	fundus	–	Type0-IIc	regular	Adenoma	NED (5)
8	M/65	Reflux	PPI	fundus	–	Type0-IIa	irregular	Adenocarcinoma	NED (33)

*HP* Helicobacter pylori, *ME-NBI* Narrow-band imaging with magnifying endoscopy, *MVP* Microvascular pattern, *NED* No evidence of disease, *NEN* Neuroendocrine neoplasm

**Table 2:** Pathological features of present cases

Patient	Tumor size (mm)	Depth of invasion (um)	mucosal atrophy/ intestinal metaplasia	Lymphatic/venous invasion	Latera/vertical margin	β-catenin	CD56/SYN/CGA	P53/CDX2/Ki67	PG-I/MUC6/MUC2/MUC5
1	8	SM(200)	−/−	−/−	−/−	+(membrane)	+/+/−	−/−	+/+/−/−
2	4	SM(100)	−/−	−/−	−/−	+(membrane)	+/+/−	−/−	+/+/−/−
3	6	MM	−/−	−/−	−/−	+(membrane)	+/+/−	−/−	+/+/−/−
4	5	SM(40)	−/−	−/−	−/−	+(membrane)	+/+/−	−/−	+/+/−/−
5	5	SM(50)	−/−	−/−	−/−	+(membrane)	+/+/−	−/−	+/+/−/−
6	12	SM(600)	+/+	−/−	−/−	+(membrane)	+/+/+	−/−	+/+/−/−
7	4	SM(100)	−/−	−/−	−/−	+(membrane)	+/+/+	−/−	+/+/−/−
8	4	MM	−/−	−/−	−/−	–	+/−/−	−/−	+/+/−/−

*PG-I* Pepsinogen-I, *MM* Muscularis mucosae, *SM* Submucosal, *Y* Years

Five of the patients presented with symptoms of gastroesophageal reflux, one presented with bloating, and two with abdominal pain that prompted endoscopic examination. Most of them were on irregular medications. Acid-suppression treatment included the use of a proton pump inhibitor (PPI) (*n* = 4) and histamine (H2) receptor antagonist (*n* = 1). The remaining patients had no history of medication. History of past illness revealed that three patients had chronic non-atrophic gastritis, but did not receive standard treatment. There was no personal and family history bearing upon the case. There was no physical examination available. None of the eight cases showed serum anti-*H. pylori* antibody in the urea breath test.

**Fig. 1:**
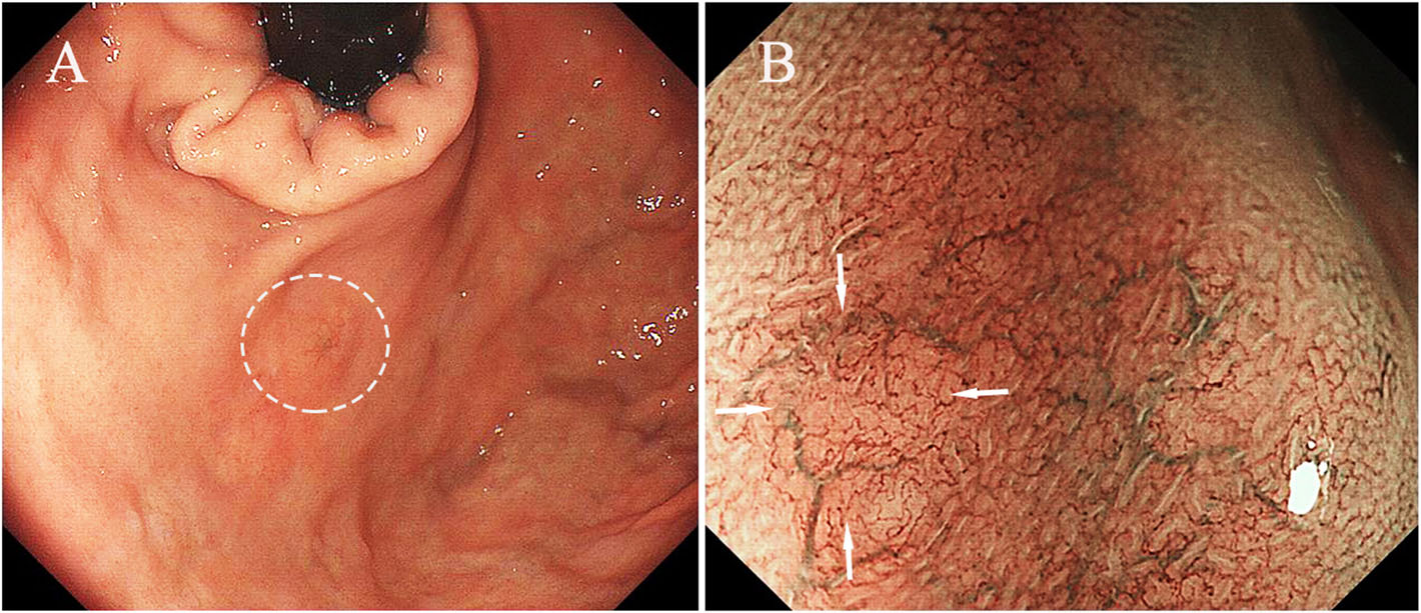
**a** Endoscopic image revealing a small depressed lesion in fundus of stomach (within circle). **b** Magnified narrow band imaging present irregular microvascular patterns with dilated vessels with branch architecture on tumor surface whereas the demarcation line was absent (arrow)

Six cases were macroscopically identified as type 0–IIa (superficial elevated type) and two were identified as type 0–IIb or c (superficial flat type or depression, respectively) (Fig. 1a). NBI-ME showed irregular MVP with dilated vessels in four cases (Fig. 1b) and regular MVP in four cases. Endoscopic examination prior to pathological diagnosis was indicative of neuroendocrine neoplasm, GA-FG, adenocarcinoma, and adenoma in two cases each.

**Fig. 2:**
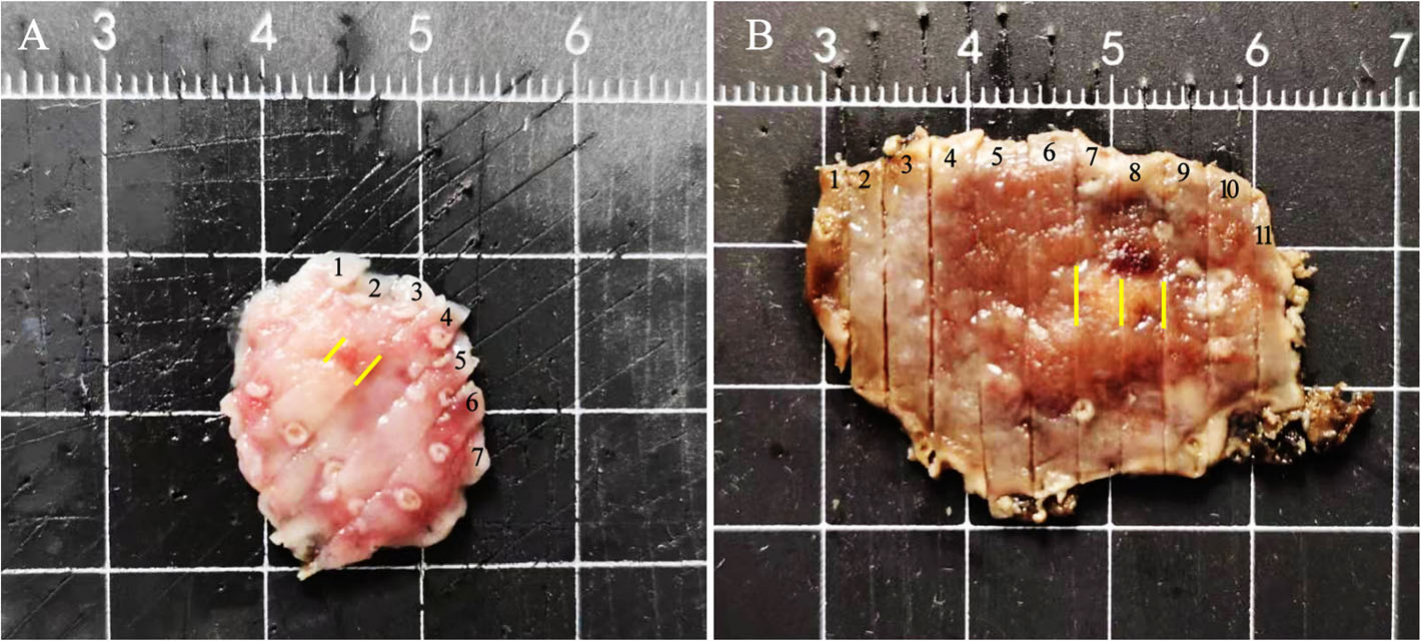
Mapping of the endoscopic submucosal dissection specimen based on histology. **a** GA-FG distributed at a slightly depression lesion measuring 5 mm × 4 mm (yellow line) (case 5); **b** showed a slightly elevated lesion measuring 12 mm × 10 mm (yellow line) (case 6)

A pathological biopsy was performed in all cases. Four cases were diagnosed as GA-FG and the remaining four as oxyntic gland adenoma owing to limitations in the depth of the mucosa layer for evaluation and the presence of well-differentiated oxyntic glands. ESD-resected specimens were subjected to conventional histological testing and immunohistochemical staining. Grossly, they were described as solitary and ranged in size from 4 to 12 mm (mean, 6 mm) (Fig. 2a, b).

**Fig. 3:**
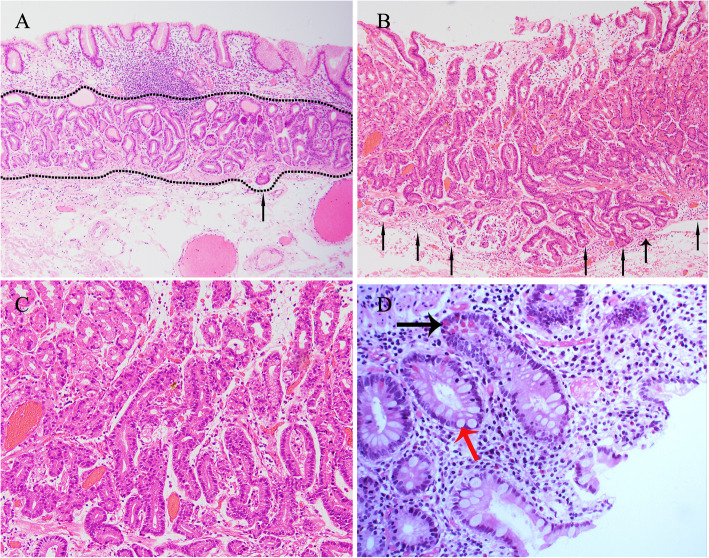
**a** Low-power view, the tumor arose from the deep layer of the lamina propria mucosa (black line) and slightly invaded the submucosal layer (black arrow), the tumor surface was covered with normal foveolar epithelium (100×). **b** Tumor cells consisted of irregular anastomosing cords and cribriform glands that are similar to the fundic glands with invasion into the submucosal layer (arrow) (100×). **c** The complex glandular architecture seen at higher magnification producing anastomosing and so-called “endless glands” pattern, the tumor cells were mild atypia (200×). **d** Intestinal metaplasia had been seen in the background, in which we can see goblet cells (red arrow) and Pan’s cells (black arrow) (case 5) (200×)

**Fig. 4:**
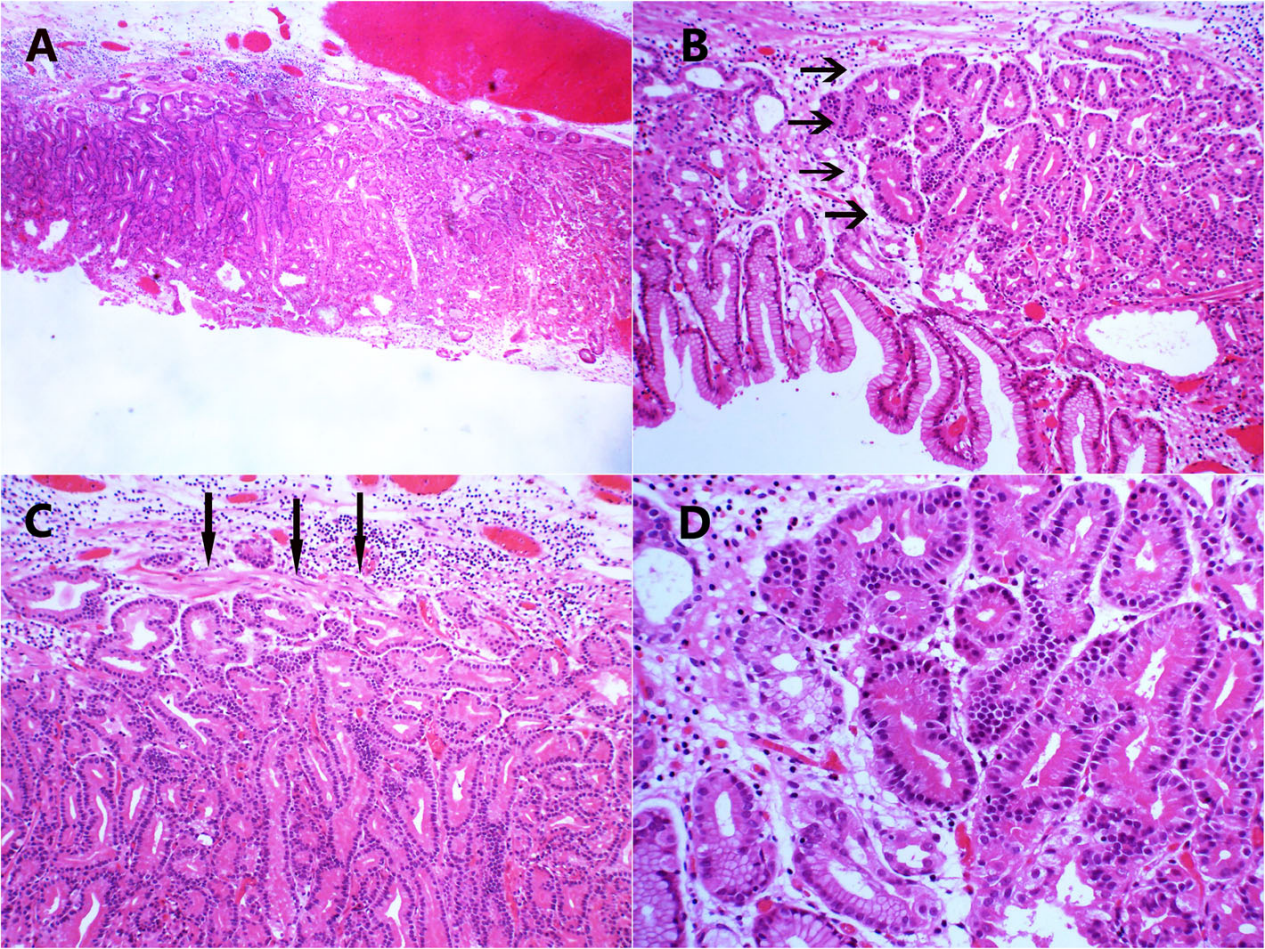
**a** Low-power view, the glands of deep mucosa layer are congested (40×). **b** The lesion had obvious transitional area with the surrounding glands (100×) (arrow).**c** the gland structure was complex and similar to the fundic glands, tumor cells broke through muscularis mucosae and invaded into submucosa (200×) (arrow). **d** The atypia of tumor cells was mild (200×)

Histologically, all tumors arose from the deep mucosa layer with an infiltrative growth pattern and most of the tumor surface was covered with normal foveolar epithelium (Fig. 3a). Six of the lesions extended into the submucosa and the depth of invasion ranged from 50 to 600 μm (average, 182 μm). The remaining cases showed partial invasion into the muscularis mucosae. In all eight present cases, chief cells were the predominant cell type. Most tumors consisted of well-formed glands of oxyntic epithelial cells; few tumor cells consisted of clustered glands, irregular anastomosing cords, and cribriform glands (Fig. 3b). Tumor cells showed mild atypia (Fig. 3c). Atrophy and intestinal metaplasia were observed in the background of one case (Fig. 3d, case 5). In case 6, the lesion arose from the deep mucosa layer with increased gland density (Fig. 4a). The lesion had obvious transitional area in the surrounding gland (Fig. 4b). The gland structure was complex, and the tumor cells had broken through the muscularis mucosae and invaded the submucosa (Fig. 4c), and the cells showed mild atypia (Fig. 4d). In all cases, lymphatic or venous invasion and lateral or vertical margin invasion were distinctly absent.

**Fig. 5:**
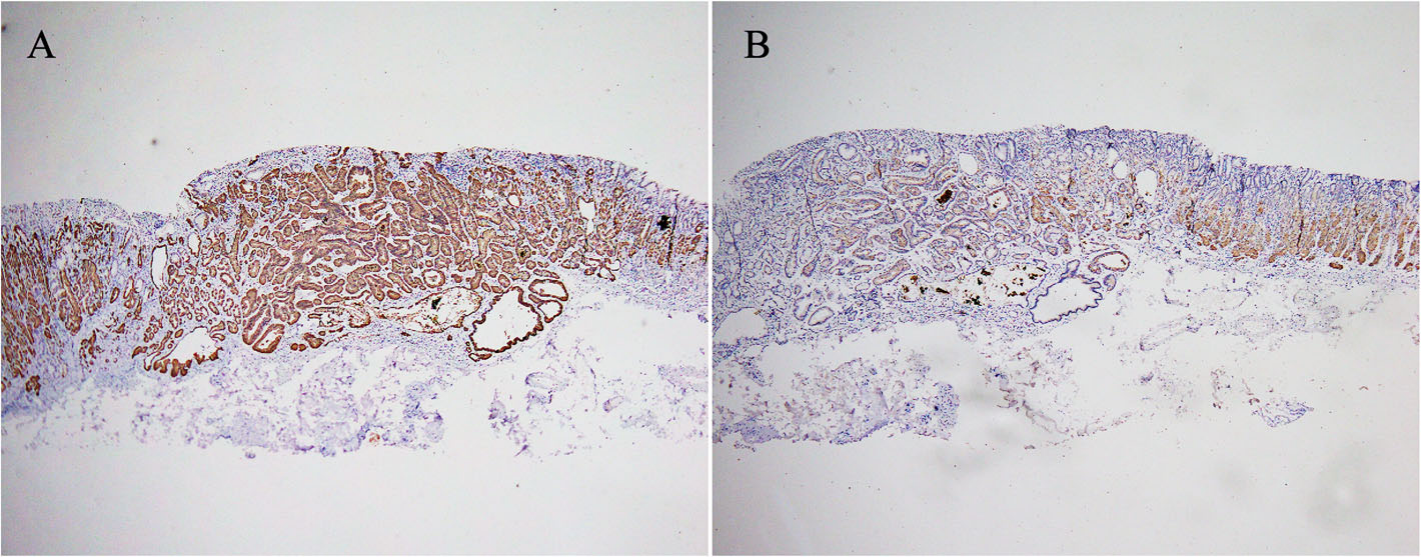
MUC6 (**a**) and Pepsinogen I (**b**) stain were strongly positive within the tumor cells confirming chief cell differentiation

**Fig. 6:**
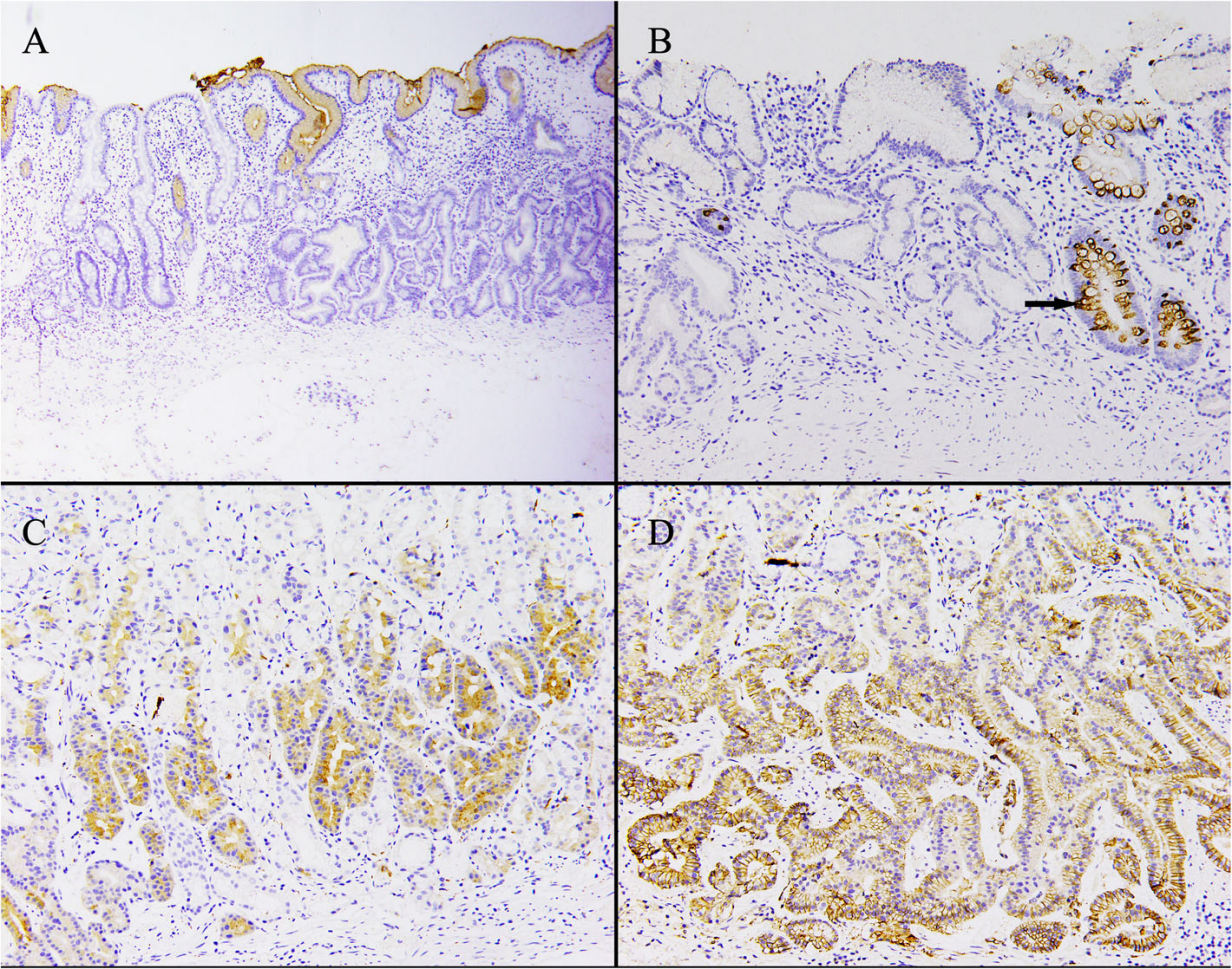
Immunohistochemical studies revealed that **a** MUC5 was negative in all tumor cells, but was positive in foveolar cells. **b** MUC2 was positive in the gland with intestinal metaplasia(arrow). Tumor cells were positive for SYN (**c**) and CD56 (**d**)

Immunohistochemical examination revealed diffuse positivity for MUC6 and pepsinogen-I for all tumors (100%) (Fig. 5a, b). In contrast, MUC2, MUC5, and CDX2 expression were negative in all cases. However, foveolar cells showed MUC5 positivity, which indicated the presence of intact foveolar epithelium (Fig. 6a). MUC2 positivity was observed in the glands with intestinal metaplasia (Fig. 6b). Consistent with morphology, CDX2 and P53 were not overexpressed and the Ki-67 labeling index was low (< 2%, with a minimum of 1000 evaluated cells). It is noteworthy that synaptophysin and CD56 positivity was observed in all eight cases (Fig. 6c, d), whereas chromogranin A positivity was scattered (two of eight). Membrane staining of β-catenin was observed with no nuclear accumulation in any of the cases.

All cases were finally diagnosed as gastric adenocarcinoma of the fundic gland type (chief cell-predominant) on the basis of endoscopy, morphology, and immunohistochemical features.

After the lesions were completely removed using ESD, patients were provided appropriate symptomatic therapy to suppress acid secretion and hemostasis, and to protect the gastric mucosa.

Follow-up examinations were performed for all patients after ESD until recently. Clinical follow-up information was available for all (100%) patients and ranged from 5 to 33 months (mean, 17 months). Disease progression or metastases were not reported.

## Discussion

Clinicopathologically, seven out of eight (7/8) GA-FGs in this study were located in the upper third and only one (1/8) tumor was observed in the middle third of the stomach. Macroscopic features indicated flatly elevated (0-IIa) (6/8) or depressed (0-IIc) lesions (2/8), and a central depression was observed in some cases with deep infiltration. This central depression was believed to be evidence of submucosal involvement [11]. Therefore, more samples should be collected to clarify whether the case with submucosal infiltration has special endoscopic features. The tumors were small with a maximum diameter ranging from 4 to 12 mm (average 6 mm). Previously published reviews showed that the average tumor diameter was 7.5 mm. Approximately 80% of all tumors were less than 10 mm in diameter at the time of diagnosis [6] and the diameter of the largest reported tumor was 85 mm [8]. Our observations were consistent with those of previous reports (Table 1).

Careful pathological observation revealed that the superficial area of the lesions almost invariably tended to retain normal foveolar epithelium, whereas the lamina propria and submucosa tended to show irregular branching and dilatation of fundic glands. The nuclei were slightly larger than those of normal fundic glands and markedly hyperchromatic. A review showed that most GA-FGs were confined to the mucosa [4]. Six of the eight (75%) cases exhibited submucosal invasion despite the small size of the lesions; lymphatic or venous invasion was not observed. Singhi et al. [10] suggested that “GA-FG” is an exaggeration and lesions should be considered benign owing to the lack of recurrence or progression. A review of 111 reported cases revealed that 57% GA-FG showed submucosal invasion, while 6% showed subserosal invasion due to lymphovascular spreading [4]. In the patients in our study, the mildly atypical glands were well-circumscribed with an abrupt transition from the normal mucosa, which is one of the signs of neoplasia. Ueyama et al. [7] speculated that surface mucosal epithelial cells are maintained, as tumors barely destroy the surrounding tissue. GA-FGs may possibly grow vertically into the submucosa and develop laterally toward the surrounding tissue. The adjacent oxyntic mucosa is normal without any intestinal metaplasia or atrophy [7]. However, a case of GA-FG arising from gastric mucosa with atrophic changes and intestinal metaplasia was focally observed in the surrounding mucosa [12]. In our study, most of the adjacent tissues of the tumor did not show inflammation, intestinal metaplasia, and atrophy, except in the case of one patient, where obvious intestinal metaplasia and atrophy were observed. Thus, this lesion is uncommon and does not invariably lack atrophy and intestinal metaplasia. Most cases, including those we evaluated, were instances of solitary tumors. Cases of multiple GA-FG have been rare, and most of their clinicopathological characteristics were similar to those seen in single lesions [5].

Immunohistochemistry showed that tumor cells diffusely expressed pepsinogen -I and MUC6, which suggested that GA-FG originated from the chief cell of the mucosal layer rather than the foveolar cells of the epithelium. All patients in our study were positive for CD56 and synaptophysin and most were negative for chromogranin A. These findings could have resulted in an incorrect diagnosis of neuroendocrine tumors. In previous studies, staining for synaptophysin and CD56 showed diffuse positivity in the glands, while chromogranin A staining revealed completely negative results [9, 11]. Since foregut-derived endocrine cells are invariably positive for chromogranin A (CGA), they certainly undergo endocrine differentiation [13–15]. Therefore, immunohistochemical findings for pepsinogen-I and MUC6 are useful for the differential diagnosis of gastric adenocarcinoma of the fundic gland type (chief cell predominant type), especially when CGA is also positive.

The differential diagnosis of GA-FG also includes the presence of neuroendocrine tumors, pyloric gland adenoma, oxyntic gland adenoma, and well-differentiated GA. Endoscopic findings may resemble those of submucosal tumor (SMT)-like tumors, especially sporadic neuroendocrine tumors [11]. However, most neuroendocrine tumors are small, smooth, firm, and well-circumscribed polypoid elevations of the mucosa and submucosa. Furthermore, Fukatsu [11] suggested that as neuroendocrine tumors grow, they may involve the entire thickness of the gastric wall, resulting in occasional central ulceration. The minute flat, elevated lesion did not reveal any polypoid appearance in GA-FG and can be useful in distinguishing GA-FG from gastric neuroendocrine tumors. A study showed similarities in the IHC profile and molecular phenotype, suggesting that GA-FG and pyloric gland adenoma may be closely related [16] and hence, may be distinguished primarily based on morphological features. Pyloric gland adenomas (PGA) show polypoid proliferation of pyloric-type glands consisting of cuboidal/columnar cells with foamy ground-glass cytoplasm. The well-differentiated (“crawling”) gastric adenocarcinoma with foveolar and pyloric phenotypes showed mild cytology and were similar to GA-FG; however, cells lacked the admixture of chief or parietal cells and showed an invasive growth pattern [17, 18]. Oxyntic gland adenoma is a benign epithelial neoplasm composed of columnar cells that can differentiate to chief cells, parietal cells, or both, and usually lacks the complex architecture of glands and submucosal invasion. Chan [9] suggested that oxyntic gland polyp/adenoma and GA-FG showed a morphological continuum, and that the adenoma was a precursor to chief cell-predominant adenocarcinoma. Whether a subset that lacks the ability to invade or metastasize can be called an “oxyntic gland adenoma” requires further investigation.

In a previous study, nuclear β-catenin positivity (using IHC) was observed in 22 of 26 cases, and 13 cases (50%) harbored mutations in at least one of the following genes: *CTNNB1*, *GNAS*, *AXIN1* or *2*, and *APC* [8]. Nuclear β-catenin expression coincided with the presence of *GNAS* mutations in four of five cases, suggesting a role for *GNAS* activation in WNT signaling. Activation of the WNT-β-catenin signaling pathway is believed to be involved in tumorigenesis [8]. Interestingly, sporadic fundic gland polyps also show activating mutations in β-catenin [19]; however, *GNAS* mutations are either absent or infrequent in conventional gastric adenomas and adenocarcinomas [20, 21]. Although only membrane staining for β-catenin without any nuclear staining was observed in all patients in our study, our results were consistent with those of Benedict et al. [4], which may be attributed to the limited number of cases.

Among the cases with follow-up data, one patient died of carcinomatosis [22] and three showed disease recurrence [10, 23], which may have been due to incomplete excision of the lesion. Complete surgical excision and fundectomy for some cases [24, 25], and ESD or EMR for most cases appear to be adequate and may lead to remission. In our study, all patients could be followed up after ESD for a period from 5 to 33 months. All patients have been free from recurrence or metastasis.

In conclusion, GA-FG is a well-differentiated adenocarcinoma with cytological mild atypia, which is often accompanied by submucosal infiltration, although lymphatic and vascular infiltration are uncommon. They are located mostly in the upper third of the stomach and composed predominantly of chief cells and known to characteristically change the complex structure of glands; however, cellular atypia is mild. The predominant immunohistochemical markers of GA-FG are pepsinogen-I and MUC6. Although not specific markers, tumor cells were invariably positive for SYN and CD56, while CGA expression was not common. The lesions were completely removed using ESD and there was no recurrence within this observation period. If GA-FG is suspected during endoscopy, a pathologist should perform immunohistochemical staining to confirm the diagnosis. More data should be collected to clarify whether acid inhibition is involved in disease development. Since GA-FG is different from conventional gastric adenocarcinoma, its etiology and pathogenesis deserve more attention.

## Data Availability

The data used and/or analyzed during the current study are available from the corresponding author on reasonable request.

## Abbreviations

GA-FG: Gastric adenocarcinoma of the fundic gland type
WHO: World Health Organization
PPI: Proton pump inhibitor
ESD: Endoscopic submucosal dissection
EMR: Endoscopic mucosal resection
NBI-ME: Narrow-band imaging with magnifying endoscopy
CGA: Chromogranin A
PG-I: Pepsinogen-I
HP: Helicobacter pylori
MVP: Microvascular pattern
NED: No evidence of disease
MM: Muscularis mucosae
SM: Submucosal
Y: Years
IHC: Immunohistochemistry
NENs: Neuroendocrine neoplasms
